# Die chirurgische Therapie der benignen Prostataobstruktion (BPO) beim antikoagulierten Patienten: eine Übersichtsarbeit über die Blutungsrisiken etablierter Techniken

**DOI:** 10.1007/s00120-020-01319-1

**Published:** 2020-09-15

**Authors:** S. Deininger, T. Herrmann, S. Schönburg, P. Törzsök, T. Kunit, L. Lusuardi

**Affiliations:** 1 Universitätsklinik für Urologie und Andrologie, Landeskrankenhaus Salzburg, Paracelsus Medizinische Privatuniversität Salzburg, Salzburg, Österreich; 2 Urologische Klinik Spital Thurgau, Kantonsspital Frauenfeld, Thurgau, Schweiz; 3 Universitätsklinik und Poliklinik für Urologie, Universitätsklinikum Halle (Saale), medizinische Fakultät, Martin-Luther-Universität Halle-Wittenberg, Halle-Wittenberg, Deutschland

**Keywords:** LUTS, Gerinnungsstörung, TURP, Vaporisation, Enukleation, LUTS, Coagulopathy, TURP, Vaporization, Enucleation

## Abstract

**Hintergrund:**

Bei einer alternden Gesellschaft ist mit einer Zunahme der therapiebedürftigen benignen Prostataobstruktion (BPO) zu rechnen, auch der Anteil an Patienten mit kardiovaskulären Komorbiditäten unter Antikoagulation steigt an. Hier kann die operative Therapie der BPO problematisch sein. Die blutstillende Wirkung der Techniken ist von besonderer Bedeutung.

**Fragestellung:**

Die folgende Übersichtsarbeit diskutiert die Datenlage zum Blutungsrisiko etablierter operativer Techniken und die Stellungnahme der EAU-Leitlinie „EAU Guidelines on Management of Non-Neurogenic Male Lower Urinary Tract symptoms (LUTS) incl. Benign Prostatic Obstruction (BPO)“ zu diesem Thema.

**Material und Methoden:**

Es wurde eine Datenanalyse aus PubMed erstellt.

**Ergebnisse:**

Die EAU-Leitlinie gibt der transurethralen Vaporisation der Prostata mittels Greenlight‑, Thulium- oder Diodenlaser und der Enukleation mittels Holmium- oder Thuliumlaser bei diesem Patientengut den Vorzug. Die bipolare ist der monopolaren transurethralen Resektion der Prostata (TURP) bei der Blutstillung überlegen. Die bipolare Enukleation der Prostata (BipoLEP) kann bei guter Hämostase zukünftig eine Alternative darstellen. Neuere minimal-invasive Techniken wie Urolift® (NeoTract, Pleasanton, USA), I‑TIND© (MediTate, Or-Akiva, Israel) und Rezūm™ (Boston Scientific, Boston, USA) zeigen ein geringes Blutungsrisiko, dies gilt auch für die Prostataarterienembolisation. Nicht geeignet erscheint die Aquaablation/AquaBeam® bei häufigen Nachblutungen. Die chirurgische Adenomektomie kann mit hoher Blutungsgefahr verbunden sein.

**Schlussfolgerung:**

Transurethrale Laservaporisation und -enukleation der Prostata sind die Therapie der Wahl beim antikoagulierten Patienten, jedoch haben auch andere transurethrale Techniken, wie die BipoLEP, ein akzeptables Blutungsrisiko und können je nach lokalen Ressourcen eine Alternative darstellen. Neuere minimal-invasive Ansätze können zukünftig mehr an Bedeutung gewinnen.

## Abstract (en)

**Background:**

In an aging society an increase of benign prostatic obstruction (BPO) requiring treatment is to be expected and the proportion of patients with cardiovascular comorbidities under anticoagulation is also increasing. As the operative treatment of BPO can be problematic, the hemostatic effect of the techniques is of particular importance.

**Objective:**

This review article discusses the data situation on the bleeding risk of established surgical techniques and the statement of the European Association of Urology (EAU) guidelines “EAU guidelines on management of non-neurogenic male lower urinary tract symptoms (LUTS), incl. benign prostatic obstruction (BPO)” on this topic.

**Material and methods:**

Data analysis from PubMed.

**Results:**

The EAU guidelines favor transurethral laser vaporization of the prostate using “greenlight”, thulium or diode laser and laser enucleation using a holmium or thulium laser in this patient collective. The bipolar is superior to monopolar transurethral resection (TUR-*P*) in hemostasis. In the future bipolar enucleation of the prostate (BipoLEP) can be an alternative under good hemostasis. Bleeding is a rare complication after recently established minimally invasive techniques, such as Urolift®, I‑TIND© and Rezum™, the same applies to prostate artery embolization. Aqua-ablation/AquaBeam® seems to be unsuitable due to frequent hematuria. Surgical adenomectomy can be associated with a high risk of bleeding.

**Conclusion:**

According to current data, transurethral laser vaporization and enucleation of the prostate are the treatment of choice for patients under anticoagulation; however, other transurethral techniques, such as BipoLEP have an acceptable risk of bleeding and can be an alternative depending on local resources. Newer minimally invasive approaches could become more important in the future.

## Einleitung

Die benigne Prostataobstruktion (BPO) zählt in der urologischen Versorgung zu den am häufigsten präsentierten Beschwerden [22]. Das Alter ist ein unabhängiger Risikofaktor für die Prävalenz und den Ausprägungsgrad [14]. In einem alternden Patientenkollektiv nimmt die Zahl derer zu, die eine Therapie mit Antikoagulantien (AC) oder Thrombozytenaggregationshemmern (TAH) benötigen. Die Indikation für eine orale AC umfasst neben Vorhofflimmern (VHF), venösen thromboembolischen Ereignissen und mechanischem Herzklappenersatz eine Vielzahl anderer thrombotischer Erkrankungen. Bei VHF ermöglicht der CHA_2_DS_2_-VASc-Score die Risikoeinstufung des Patienten und die Entscheidung, ob eine Thromboembolieprophylaxe indiziert ist (Tab. 1; [17]). Davon abzugrenzen sind TAH, welche u. a. bei Myokardinfarkt, Schlaganfall und peripherer arterieller Verschlusskrankheit eingesetzt werden.

Anders als beim Themenkomplex „antegrade Ejakulation“ bleibt bei der Behandlung der antikoagulierten Patienten der primäre Endpunkt „symptomatische und objektivierbare Verbesserung der Miktionsparameter“ unverändert. Der chirurgischen Sicherheit, die auch sonst ein Qualitätsmarker für transurethrale Eingriffe darstellt, gilt hierbei ein besonderes Augenmaß.

Die chirurgische BPO Therapie beim antikoagulierten Patienten ist eine besondere Herausforderung

Der folgende Artikel behandelt die in der Klinik etablierten operativen Therapiemöglichkeiten der BPO, das Blutungsrisiko und die Datenlage bei Patienten unter AC und TAH in Bezugnahme auf die Empfehlungen der EAU-Leitlinie „EAU Guidelines on Management of Non-Neurogenic Male Lower Urinary Tract symptoms (LUTS) incl. Benign Prostatic Obstruction (BPO)“ [13].

**Table 1:** 

		Punkte
C	„*C*ongestive heart failure“ (Herzinsuffizienz)	1
H	*H*ypertonie	1
A_2_	*A*lter ≥75 Jahre	2
D	*D*iabetes mellitus	1
S_2_	*S*chlaganfall/TIA/Thromboembolie	2
V	Kardio*v*askuläre Erkrankung (Myokardinfarkt, pAVK, Aortenplaque)	1
A	*A*lter 65–74 Jahre	1
Sc	„*S*ex *c*ategory“ (Geschlecht weiblich)	1

*pAVK* periphere arterielle Verschlusskrankheit

## Endoskopisch transurethrale Techniken

### Transurethrale strombasierte Techniken zur Resektion und Vaporisation

#### Monopolare transurethrale Resektion (M-TURP) und Inzision der Prostata (M-TUIP)

Die M‑TURP gilt als Standardtherapie der BPO bei Prostatavolumina von bis zu 80 g [13]. Sie verbessert signifikant den Harnstrahl (Q_max_), die Gesamtpunktzahl des International Prostate Symptom Score (IPSS), die Lebensqualität (QoL) aus dem IPSS und die Restharnmenge, die Reoperationsrate ist gering. Eine der häufigsten Komplikationen ist die Blutung: bei bis zu 4,9 % der Patienten tritt eine Blasentamponade auf, bis zu 9 % benötigen eine Bluttransfusion (BT, [8]). Bei antikoagulierten Patienten ist v. a. die Zahl an Blasentamponaden höher: Michielsen et al. [20] zeigten 2011 an einer Kohorte von 78 Patienten unter AC(Antikoagulantien)/TAH(Thrombozytenaggregationshemmer) einen durchschnittlichen Hb-Verlust von 1,21 ± 0,92 mg/dl nach M‑TURP, 2,6 % der Patienten benötigten eine BT, und bei 15 % trat eine Blasentamponade auf.

#### Bipolare transurethrale Resektion (B-TURP), Vaporisation (B-TUVP) und Enukleation der Prostata (BipolEP)

Im Vergleich zur M‑TURP scheint die B‑TURP besser hämostyptisch wirksam zu sein: Eine Metaanalyse von Mamoulakis et al. [19] 2009 mit 1406 Patienten ergab eine geringere Rate an Blasentamponaden (p* = 0,03) und an postoperativer Spülungsbedürftigkeit (p* < 0,00001) nach B‑TURP im Vergleich zu M‑TURP, ohne Unterschied in der Rate an BT. Auch bei Patienten unter AC/TAH scheint die B‑TURP ein vertretbares Risikoprofil zu besitzen: El-Shaer et al. [10] zeigten 2017 bei 91 voll antikoagulierten Patienten einen durchschnittlichen perioperativen Hämoglobin- (Hb‑)Verlust von 0,74 g/dl, mit einer Rate an Blasentamponaden von 2,2 % und von BT von 2,2 %. Auch die postoperativ funktionellen Ergebnisse waren mit denen der M‑TURP vergleichbar.

Die BipoLEP stellt eine sichere endoskopische Operationstechnik mit geringem Blutungsrisiko dar

Die plasmakinetische B‑TUVP ist eine Alternative der B‑TURP, bei der das Adenomgewebe vaporisiert wird. Im Vergleich zur TURP zeigt sich hier ein geringerer Hb-Verlust und subjektiv reduzierte intraoperative Blutung [12]. Die EAU-Leitlinie sieht die B‑TUVP als Alternative zur M‑TURP [13]. Eine weitere Variante der B‑TURP ist die bipolare Enukleation der Prostata (BipolEP), bei der das Adenomgewebe mittels bipolarer Schlinge enukleiert und in der Blase morcelliert wird. Die Datenlage zur BipoLEP ist aktuell eingeschränkt, insbesondere liegen wenige Ergebnisse zur Sicherheit bei Patienten unter AC/TAH vor [16, 21].

Eine Metaanalyse von Arcaniolo et al. [3] aus 2019 zeigt eine Überlegenheit der BipoLEP über die B‑TURP nicht nur hinsichtlich der Dauer von Katheterisierung (p* = 0,006) und Krankenhausaufenthalt (p* < 0.0001), sondern auch bei Hb-Verlust (p* = 0,03), bei kurz- und langfristigen Komplikationen sowie bei verschiedenen Parametern zum funktionellen Outcome. Sicherlich sind weitere randomisiert kontrollierte Studien (RCT) notwendig, um die Sicherheit der BipoLEP auch bei Patienten unter AC/TAH zu prüfen, die ersten Ergebnisse sind jedoch vielversprechend und die BipoLEP könnte zukünftig eine Alternative zu den transurethralen laserbasierten Techniken darstellen.

### Transurethrale laserbasierte Techniken zu Vaporisation, Enukleation und Inzision der Prostata

#### Holmium:Yttrium-Aluminium Garnet- (Ho:YAG-)Laservaporisation, -enukleation (HoLEP) und -inzision (Ho-TUIP)

Mittels Holmiumlaser wird die Prostata bevorzugt enukleiert (HoLEP), die Ho-TUIP stellt eine Minimalvariante dar. Bei HoLEP zeigt sich im Vergleich zu TURP ein geringerer Blutverlust (*p = 0,001; [29]). Auch bei Patienten unter AC/TAH konnten Boeri et al. [5] 2019 HoLEP sicher durchführen: Im Vergleich zur nicht-antikoagulierten Kontrollgruppe hatten die Patienten mit AC/TAH eine länger andauernde Phase von Katheterversorgung (*p < 0,01) und Krankenhausaufenthalt (p* < 0,01), ansonsten ergab sich kein Unterschied im postoperativen Outcome.

#### Greenlight- (532 nm)/Laservaporisation (photoselektive Vaporisation, PVP) und -enukleation der Prostata (GreenLEP)

Die funktionellen Ergebnisse nach PVP hinsichtlich der Verbesserung von Q_max_ und IPSS sind denen nach TURP vergleichbar, die Rate an perioperativer BT ist niedriger (p* < 0,00001; [8]). Größere RCT zum Thema PVP bei antikoagulierten Patienten fehlen, es gibt lediglich Fallserien. Ruszat et al. [26] zeigten 2007 bei 116 Männern unter AC/TAH im Vergleich zur nicht antikoagulierten Kontrollgruppe keinen Unterschied in Operationsdauer, Hb-Verlust und bei den funktionellen Ergebnissen. Die EAU-Leitlinie sieht die PVP zusammen mit ThuVEP als erste Wahl bei Patienten unter AC/TAH und einem Prostatavolumen unter 80 g [13]. Die GreenLEP ist insgesamt wenig untersucht, kleine Studien legen jedoch auch ein akzeptables Sicherheitsprofil nahe [24].

#### Thulium:Yttrium-Aluminium-Garnet-Laser- (Tm:YAG‑)Vaporisation (ThuVAP), -Vaporeserektion (ThuVARP), -Vapoenukleation (ThuVEP) und Tm:YAG-laserassistierte anatomische Enukleation (ThuLEP) der Prostata

Sowohl ThuVARP als auch ThuVEP scheinen, was die funktionellen Ergebnisse angeht, mit den Standardtechniken vergleichbar zu sein [30]. Daneben besitzt der Tm:YAG-Laser ein akzeptables Risikoprofil hinsichtlich perioperativen Blutungskomplikationen, auch bei Patienten mit AC/TAH.

Hauser et al. [15] untersuchten 2011 ThuVEP bei 39 Patienten mit AC/TAH und oder Gerinnungsstörungen. Der mediane perioperative Hb-Abfall lag bei 1,2 g/l, 2,6 % der Patienten erhielten eine BT (2,6 %), 12,8 % litten im Verlauf unter leichtgradiger HU (12,8 %). Die EAU Leitlinie nennt ThuVEP und ThuVARP als Alternativen zur TURP bei Patienten unter AC/TAH [13].

Eine Variante der ThuVEP stellt die ThuLEP dar, bei der das Adenom stumpf abpräpariert und der Laser lediglich zur Inzision der Mukosa und zur Durchtrennung von Adhäsionen sowie zur Blutstillung verwendet wird [16]. Erwartbar wäre so eine gute Hämostase durch die anatomische Enukleation, im Rahmen einer Metaanalyse zeigten Xiao et al. [28] 2019 einen Trend zum geringeren Hb-Verlust bei ThuLEP im Vergleich zu HoLEP, jedoch ohne statistische Signifikanz.

#### Diodenlaservaporisation (DiVAP) und -enukleation (DiLEP)

Die funktionellen Ergebnisse nach DiVAP [13] und DiLEP sind v. a. im kurzfristigen Verlauf denen der TURP vergleichbar, mit geringerem intraoperativen Blutverlust und Dauer von Klinikaufenthalt und Katheterisierung nach DiLEP [18]. Die Datenlage legt gute hämostyptische Eigenschaften des Diodenlasers nahe: nach DiVAP an 55 Patienten, von denen 23,6 % AC/TAH einnahmen, benötigte bei Chiang et al. [6] 2010 keiner der Patienten eine BT oder eine elektrische Nachkoagulation.

### Sonstige transurethrale Techniken

#### Das Urolift®-Implantat

Das permanente Urolift®-Implantat (NeoTract, Pleasanton, USA) führt zu einer signifikanten Verbesserung von IPSS, QoL und Q_max_, ohne retrograde Ejakulation zu bedingen [11]. Nicht untersucht wurden bis jetzt die Rate an Reoperationen, die möglichen Komplikationen oder die Anwendung bei Patienten unter AC/TAH, aufgrund dessen empfiehlt die EAU-Leitlinie die Implantation eines Urolift®-Systems nur bei gut aufgeklärten Männern, welche am Erhalt ihrer Sexualfunktion interessiert sind [13].

#### Das „temporary implantable nitinol device“: das I-TIND©-Implantat

Das temporäre I‑TIND©-Implantat (MediTate, Or-Akiva, Israel) verursacht eine Rinnenbildung vergleichbar einer Turner-Warwick-Inzision und verbessert IPSS, QoL und Q_max_ signifikant. Komplikationen treten selten auf, insbesondere wurden keine Blutungen beobachtet [23]. Es existieren keine Daten zur I‑TIND©-Therapie bei Patienten unter AC/TAH. Die EAU-Leitlinie [13] gibt aufgrund der fehlenden Daten keine Empfehlung hinsichtlich der Verwendung ab.

#### Die konvektive Wasserdampfablation WAVE™: das Rezūm™-System

Bei der konvektiven Wasserdampfablation wird heißer Wasserdampf in das Adenom geleitet, was zur Nekrose der Adenomzellen führt. Im Rahmen einer RCT an 197 Männern konnte Rezūm™ (Boston Scientific, Boston, USA) IPSS und Q_max_ nach 3 Monaten signifikant reduzieren. Insgesamt scheint das Komplikationsrisiko der Rezūm™-Therapie gering zu sein, Blutungskomplikationen werden mit maximal 13,8 % HU (Clavien Dindo maximal II) beschrieben [25]. Der Hersteller selbst empfiehlt das Pausieren von Cumarinen für 3, von TAH für 7 Tage präoperativ. Die EAU-Leitlinie spricht keine Empfehlung zur oder gegen die Anwendung von Rezūm™ aus [13].

#### Aquaablation: das AquaBeam®-System

Bei der Aquaablation (AquaBeam®, Procept Biorobotics, Redwood City, Kalifornien, USA) wird das Adenomgewebe unter rektaler Ultraschallkontrolle computergesteuert mittels eines Kochsalzwasserstrahls abgetragen. Blutstillung wird abschließend mittels Kompression über einen transurethralen Dauerkatheter oder über Laserkoagulation erreicht. Manche der Autoren führen inzwischen regelhaft eine abschließende transurethrale Koagulation durch.

Die Datenlage zeigt gute funktionelle Ergebnisse vergleichbar der TURP. Im Rahmen des RCT *WATER II* zeigte sich jedoch ein erhöhtes Blutungsrisiko: 13,9 % der Patienten erlitten Nachblutungen, 7,9 % erhielten eine BT, 3 % mussten transurethral operativ revidiert werden, und 2 % benötigten sowohl operative Revision als auch BT [9]. Die Aquaablation scheint sich anhand der aktuellen Datenlage nicht für Patienten unter AC/TAH anzubieten, die EAU-Leitlinie stuft die Aquaablation als Alternative zur TURP mit jedoch erhöhtem Blutungsrisiko ein [13].

## Prostataarterienembolisation (PAE)

Im Rahmen der PAE werden über arteriellen Zugang und mittels digitaler Subtraktionsangiografie (DSA) selektiv die Arterien der Prostata embolisiert, wodurch sich IPSS, QoL, Q_max_ und Restharnbildung signifikant verbessern [4].

Präoperativ geprüft wird die Dringlichkeit der OP, und ob die AC/TAH pausiert werden kann

Im Vergleich zur TURP ist der perioperative Blutverlust geringer, bei jedoch vergleichsweise schlechterem funktionellen Ergebnis [1]. Die PAE ist bei Patienten unter AC/TAH nicht ausreichend untersucht. Andere interventionelle Techniken wie die Koronarangiographie, deren Zugangsweg und Invasivität mit denen der PAE vergleichbar erscheinen, legen jedoch ein akzeptables Sicherheitsprofil solcher Therapieverfahren nahe [2]. Die EAU-Leitlinie empfiehlt die PAE Männern, welche an minimal-invasiven operativen Ansätzen interessiert und bereit sind, ein vergleichsweise schlechteres funktionelles Ergebnis zu akzeptieren [13].

## Offene (OP) oder roboterassistierte Prostatektomie (RAP)

Die wohl invasivste Methode der operativen BPO-Therapie ist die offene oder roboterassistierte laparoskopische Prostatektomie. Die perioperativen Blutungsrisiken sind nicht zu unterschätzen: bei bis zu 27 % der Fälle ergibt sich die Notwendigkeit einer BT [7]. Unserem Kenntnisstand nach gibt es keine Daten über die Sicherheit von Operation bei antikoagulierten Patienten. Die EAU-Leitlinie empfiehlt die offene Prostatektomie aufgrund des deutlichen Risikoprofils nur bei Männern mit Prostatavolumina über 80 g, wenn nicht die Möglichkeit einer transurethralen Enukleation besteht [13].

## Patientenselektion: Empfehlungen zur Antikoagulation aus den EAU-Leitlinien

Laut aktueller EAU-Leitlinie soll bei antikoagulierten Patienten geprüft werden, ob die Antikoagulation perioperativ gestoppt werden kann. Ist dies der Fall, empfehlen sich Standardtechniken je nach Prostatavolumen. Eine Empfehlung dazu, ob das Pausieren der AC/TAH möglich ist, liefert die EAU-Leitlinie „Thromboprophylaxis“. Folgendes Vorgehen wird vorgeschlagen: AC und TAH sollen ersatzlos perioperativ abgesetzt und nach ca. 4 Tagen erneut begonnen werden, sobald das Blutungsrisiko vertretbar ist. Eine Ausnahme bildet ein unaufschiebbarer Eingriff an folgenden Risikopatienten: Drug-eluting-stent-Implantation <6 Monate, Bare-metal-stent-Implantation <6 Wochen oder transient ischämische Attacke (TIA) <30 Tage. In diesem Fall soll die Medikation fortgeführt werden. Perioperativ mit niedermolekularem Heparin (NMH) gebridgt werden sollen lediglich Patienten mit schwerer Thrombophilie (Antithrombinmangel oder Antiphospholipidsyndrom) und mit mechanischen Herzklappen mit hohem thrombotischen Risiko [27]. Kann eine AC/TAH im Rahmen der genannten Konstellationen nicht pausiert werden oder muss mit NMH gebridgt werden, sollen die transurethralen Techniken Laservaporisation (beinhaltet PVP, ThuVAP und DiVAP) und -enukleation (HoLEP und ThuVEP) der Prostata zum Einsatz kommen [13].

## Zusammenfassung

**Figure 1:**
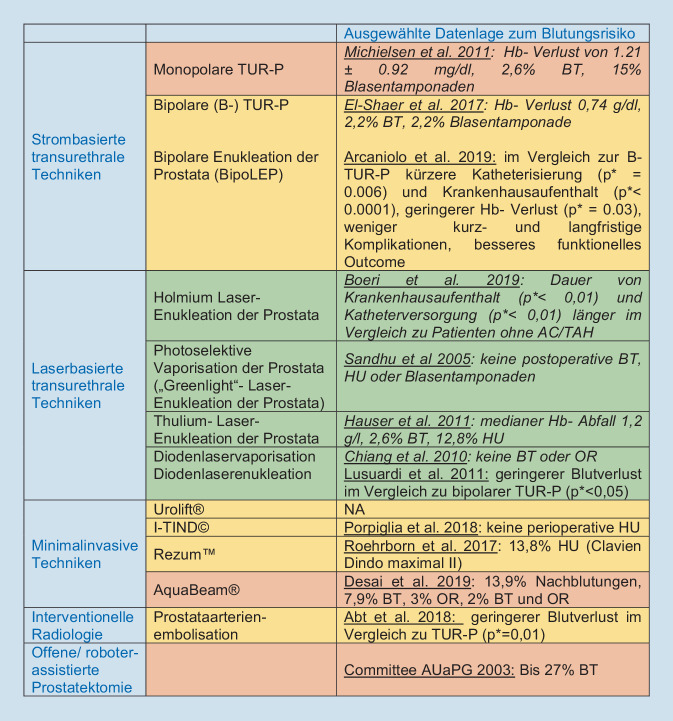

Die Urologie sieht sich die kommenden Jahrzehnte mit einem alternden Patientengut konfrontiert. Damit steigt auch die Zahl derer mit kardiovaskulären Komorbiditäten und unter Therapie mit AC/TAH. Die heute zur Verfügung stehenden operativen Techniken zur Therapie der BPO sind heterogen, haben unterschiedliche funktionelle Ergebnisse und Komplikationspotentiale. Für Patienten unter AC/TAH ist die hämostyptische Eigenschaft einer Technik entscheidend. Abb. 1 zeigt eine Zusammenfassung etablierter Techniken mit einer Auswahl an Daten zum Blutungsrisiko, sowie eine Einschätzung der Autoren, inwiefern die Technik sich für antikoagulierte Patienten eignet.

Bei der Wahl der operativen BPO-Therapie beim Patienten unter AC/TAH sollten verschiedene Punkte bedacht werden:

1. Können AC/TAH pausiert werden und wenn ja wie lange?
2. Muss mit NMH gebridgt werden?
3. Wie alt und in welchem Allgemeinzustand ist der Patient und wie wahrscheinlich ist in diesem Zusammenhang die Notwendigkeit einer Reoperation?
4. Ist das Risiko einer Narkose zumutbar?

Im Gespräch mit dem Patienten und dem betreuenden Internisten kann die Therapie nach Abwägung der Vor- und Nachteile der Techniken zusammen ausgewählt werden, auch in Zusammenschau mit den lokalen Ressourcen.

## Fazit für die Praxis

- Laut Leitlinie sollen bevorzugt transurethrale Laservaporisation (beinhaltet photoselektive Vaporisation der Prostata [PVP], Thuliumvaporisation der Prostata [ThuVAP] und Diodenlaservaporisation der Prostata [DiVAP]) und -enukleation (Holmiumlaserenukleation der Prostata [HoLEP] und Tm:YAG-laserassistierte anatomische Enukleation [ThuVEP]) der Prostata zum Einsatz kommen.
- Die bipolare (B-TURP) ist der monopolaren TURP (M-TURP) bei der Blutstillung überlegen.
- Die bipolare Enukleation der Prostata (BipoLEP) zeigt im Vergleich zur B‑TURP ein geringeres Blutungsrisiko bei gutem funktionellen Outcome, und stellt möglicherweise zukünftig eine Alternative dar.
- Neuere minimal-invasive Techniken wie Urolift® (NeoTract, Pleasanton, USA), I‑TIND© (MediTate, Or-Akiva, Israel) und Rezūm™ (Boston Scientific, Boston, USA) sind noch nicht genügend untersucht, könnten jedoch eine mögliche Therapieoption darstellen. Nicht geeignet erscheint hingegen die Aquaablation bei teilweise ausgeprägtem Nachblutungsrisiko.
- Auch die Prostataarterienembolisation (PAE) als Technik aus der interventionellen Radiologie hat ein akzeptables Blutungsrisiko.
- Offen-chirurgische oder laparoskopische Adenomektomien sind mit hohem Blutungsrisiko verbunden.

## References

[1] Abt D, Hechelhammer L, Mullhaupt G (2018). Comparison of prostatic artery embolisation (PAE) versus transurethral resection of the prostate (TURP) for benign prostatic hyperplasia: randomised, open label, non-inferiority trial. BMJ.

[2] Annala AP, Karjalainen PP, Porela P (2008). Safety of diagnostic coronary angiography during uninterrupted therapeutic warfarin treatment. Am J Cardiol.

[3] Arcaniolo D, Manfredi C, Veccia A (2020). Bipolar endoscopic enucleation versus bipolar transurethral resection of the prostate: an ESUT systematic review and cumulative analysis. World J Urol.

[4] Bhatia S, Sinha VK, Harward S (2018). Prostate artery embolization in patients with prostate volumes of 80 mL or more: a single-institution retrospective experience of 93 patients. J Vasc Interv Radiol.

[5] Boeri L, Capogrosso P, Ventimiglia E (2020). Clinical comparison of holmium laser enucleation of the prostate and bipolar transurethral enucleation of the prostate in patients under either anticoagulation or antiplatelet therapy. Eur Urol Focus.

[6] Chiang PH, Chen CH, Kang CH (2010). GreenLight HPS laser 120-W versus diode laser 200-W vaporization of the prostate: comparative clinical experience. Lasers Surg Med.

[7] Committee AUaPG (2003). AUA guideline on management of benign prostatic hyperplasia (2003). Chapter 1: Diagnosis and treatment recommendations. J Urol.

[8] Cornu JN, Ahyai S, Bachmann A (2015). A systematic review and meta-analysis of functional outcomes and complications following transurethral procedures for lower urinary tract symptoms resulting from benign prostatic obstruction: an update. Eur Urol.

[9] Desai M, Bidair M, Zorn KC (2019). Aquablation for benign prostatic hyperplasia in large prostates (80–150 mL): 6-month results from the WATER II trial. BJU Int.

[10] El-Shaer W, Abou-Taleb A, Kandeel W (2017). Transurethral bipolar plasmakinetic vapo-enucleation of the prostate: Is it safe for patients on chronic oral anticoagulants and/or platelet aggregation inhibitors?. Arab J Urol.

[11] Garcia C, Chin P, Rashid P (2015). Prostatic urethral lift: a minimally invasive treatment for benign prostatic hyperplasia. Prostate Int.

[12] Geavlete B, Georgescu D, Multescu R (2011). Bipolar plasma vaporization vs monopolar and bipolar TURP-A prospective, randomized, long-term comparison. Urology.

[13] Gravas SCJN, Gacci M, Gratzke C, Herrmann TRW, Mamoulakis C, Rieken M, Speakman MJ, Tikkinen KAO (2019). Management of non-neurogenic male lower urinary tract symptoms (LUTS), incl. benign prostatic obstruction (BPO).

[14] Haidinger G, Temml C, Schatzl G (2000). Risk factors for lower urinary tract symptoms in elderly men. For the Prostate Study Group of the Austrian Society of Urology. Eur Urol.

[15] Hauser S, Rogenhofer S, Ellinger J (2012). Thulium laser (Revolix) vapoenucleation of the prostate is a safe procedure in patients with an increased risk of hemorrhage. Urol Int.

[16] Herrmann TRW, Gravas S, De La Rosette JJ (2020). Lasers in transurethral enucleation of the prostate-do we really need them. J Clin Med.

[17] Lip GY, Nieuwlaat R, Pisters R (2010). Refining clinical risk stratification for predicting stroke and thromboembolism in atrial fibrillation using a novel risk factor-based approach: the euro heart survey on atrial fibrillation. Chest.

[18] Lusuardi L, Myatt A, Sieberer M (2011). Safety and efficacy of eraser laser enucleation of the prostate: preliminary report. J Urol.

[19] Mamoulakis C, Ubbink DT, De La Rosette JJ (2009). Bipolar versus monopolar transurethral resection of the prostate: a systematic review and meta-analysis of randomized controlled trials. Eur Urol.

[20] Michielsen DP, Coomans D, Van Lersberghe C (2011). Comparison of the haemostatic properties of conventional monopolar and bipolar transurethral resection of the prostate in patients on oral anticoagulants. Arch Med Sci.

[21] Naspro R, Gomez Sancha F, Manica M (2017). From “gold standard” resection to reproducible “future standard” endoscopic enucleation of the prostate: what we know about anatomical enucleation. Minerva Urol Nefrol.

[22] Oelke M, Bachmann A, Descazeaud A (2013). EAU guidelines on the treatment and follow-up of non-neurogenic male lower urinary tract symptoms including benign prostatic obstruction. Eur Urol.

[23] Porpiglia F, Fiori C, Bertolo R (2018). 3-Year follow-up of temporary implantable nitinol device implantation for the treatment of benign prostatic obstruction. BJU Int.

[24] Rijo E, Misrai V, Gomez-Sancha F (2019). Vapoenucleation of the prostate using 180 W green light laser. Urology.

[25] Roehrborn CG, Gange SN, Gittelman MC (2017). Convective thermal therapy: durable 2-year results of randomized controlled and prospective crossover studies for treatment of lower urinary tract symptoms due to benign prostatic hyperplasia. J Urol.

[26] Ruszat R, Wyler S, Forster T (2007). Safety and effectiveness of photoselective vaporization of the prostate (PVP) in patients on ongoing oral anticoagulation. Eur Urol.

[27] Violette PD, Cartwright R, Briel M (2016). Guideline of guidelines: thromboprophylaxis for urological surgery. BJU Int.

[28] Xiao KW, Zhou L, He Q (2019). Enucleation of the prostate for benign prostatic hyperplasia thulium laser versus holmium laser: a systematic review and meta-analysis. Lasers Med Sci.

[29] Yin L, Teng J, Huang CJ (2013). Holmium laser enucleation of the prostate versus transurethral resection of the prostate: a systematic review and meta-analysis of randomized controlled trials. J Endourol.

[30] Zhao C, Yang H, Chen Z (2016). Thulium laser resection versus plasmakinetic resection of prostates in the treatment of benign prostate hyperplasia: a meta-analysis. J Laparoendosc Adv Surg Tech A.

